# Macrophage activation syndrome in a patient with adult-onset Still’s disease following first COVID-19 vaccination with BNT162b2

**DOI:** 10.1186/s41927-021-00237-9

**Published:** 2021-12-28

**Authors:** Frédéric Muench, Martin Krusche, Leif Erik Sander, Thomas Rose, Gerd-Rüdiger Burmester, Udo Schneider

**Affiliations:** 1 Department of Nephrology and Medical Intensive Care, Charité - Universitätsmedizin Berlin, Berlin, Germany; 2 Department of Rheumatology and Immunology, Charité - Universitätsmedizin, Berlin, Germany; 3 Department of Rheumatology and Systemic Inflammatory Diseases, Universitätsklinikum Hamburg-Eppendorf, Hamburg, Germany; 4 Department of Infectious Diseases and Respiratory Medicine, Charité - Universitätsmedizin, Berlin, Germany

**Keywords:** Adult-onset Still’s disease, Macrophage activation syndrome, Vaccine reaction

## Abstract

**Background:**

Adult-onset Still’s disease (AOSD) is an autoinflammatory multi-systemic syndrome. Macrophage activation syndrome (MAS) is a potentially life-threatening complication of AOSD with a mortality rate of 10–20%. Especially viral infection is thought to be a common trigger for development of MAS. On the other hand, the occurrence of MAS following vaccinations is extremely rare and has been described in a few cases after measles or influenza vaccinations and more recently after ChAdOx1 nCoV-19 (COVID-19 viral vector vaccine, Oxford-AZ).

**Case presentation:**

We report the case of a twenty-year-old female with adult-onset Still’s disease (AOSD), who developed a MAS six days after receiving her first COVID-19 vaccine dose of BNT162b2 (mRNA vaccine, BioNTech/Pfizer) with ferritin levels of 136,680 µg/l (ref.: 13–150 µg/l).

**Conclusions:**

To the best of our knowledge, this is the first case report of development of MAS in a patient with preexisting AOSD after vaccination in general, and SARS-CoV-2 vaccination in particular. The new mRNA vaccines have generally shown a reassuring safety profile, but it has been shown that nucleic acids in general, including mRNA can act as pathogen-associated molecular patterns that activate toll-like receptors with extensive production of pro-inflammatory cytokines and further activation of immune cells. Proving an interferon 1 response in our patient directly after vaccination, we think that in this particular case the vaccination might have acted as trigger for the development of MAS. Even if it remains difficult to establish causality in the case of rare adverse events, especially in patients with autoimmune or autoinflammatory conditions, these complications are important to monitor and register, but do not at all diminish the overwhelming positive benefit-risk ratio of licensed COVID-19 vaccines.

**Supplementary Information:**

The online version contains supplementary material available at 10.1186/s41927-021-00237-9.

## Background

Adult-onset Still’s disease AOSD is an autoinflammatory multi-systemic syndrome. Macrophage activation syndrome (MAS) is a potentially life-threatening complication of AOSD, which occurs in up to 12–14% of AOSD patients and has a mortality rate of 10–20% [1]. Therefore, early detection and immediate treatment and close monitoring is required. Several factors may contribute to the progress to MAS as proposed in a multilayer pathophysiology model [2]. This includes patients’ genetics, inflammatory activity of the autoimmune condition and triggers such as infections. Together, this leads to a disturbance in immune homeostasis, with abnormal activation of immune cells, especially T cells, natural killer (NK) cells and macrophages, and an overproduction of inflammatory cytokines, which, when reaching a critical threshold, results in manifest MAS. Particularly viral infection is thought to be a common trigger for development of MAS, such as Epstein Barr Virus (EBV), other Herpes viruses or H1N1 influenza viruses, but also infection with Leishmania [3]. On the other hand, the occurrence of MAS following vaccinations is extremely rare. Some cases of MAS after measles or influenza vaccination have previously been reported in a very small number of patients [4] [5]. Recently, three cases of MAS after SARS-CoV-2 vaccination with ChAdOx1 nCoV-19 (viral vector vaccine, Oxford-AZ) and one case in China with an unknown vaccine agent were reported, though without discussion of immunological mechanisms of MAS development [6] 7. Also, two case reports of de-novo AOSD following COVID-19 vaccination have been published, one after mRNA-1273 (mRNA vaccine, ModernaTX) vaccine and one after ChAdOx1 nCoV-19 [8]. Recent case studies further described inflammatory myocarditis-like illness after vaccination with different vaccines (BNT162b2 (mRNA vaccine, BioNTech/Pfizer) as well as with mRNA-1273 and Ad.26.COV2.S (viral vector vaccine, Johnson & Johnson)), although pathophysiological hypotheses could not be established [9].

We report the case of a twenty-year-old female with AOSD, who developed a manifest MAS six days after receiving her first vaccine dose of BNT162b2.

## Case presentation

The patient was first diagnosed with AOSD in August 2020 and had experienced one relapse in February 2021 with skin rash, arthralgia, fever, liver failure and hyperferritinemia (max. 17,092 µg/l; ref.: 13–150 µg/l). At the time of SARS-CoV-2 vaccination, the patient had been in stable condition for more than three months under maintenance therapy with anakinra (2 × 100 mg/d) and prednisolone (5 mg/d).

Six days after the first vaccination, she reported severe fatigue and intermittent fever episodes (> 39.5 °C). In the following days, she experienced severe myalgia, a sore throat, nausea, tremor, sweating and dizziness. Arthralgia or skin rash were absent. Laboratory results showed highly elevated serum ferritin (136,680 µg/l), triglycerides (352 mg/dL; < 200 mg/dL), serum calprotectin / S100A8/9 (> 24 µg/l; < 2.94 µg/l), sIL-2-R (14′068 UI/ml; < 710.0 UI/ml), LDH (3′136 U/L; 135–250 U/L), CRP (46 mg/L; < 5 mg/L), CD169/SIGLEC1 expression on monocytes (5753 AG/cell; < 2400 AG/cell), liver and cholestasis parameters, signs of coagulopathy (elevated d-dimers (> 35 mg/L; < 0.5 mg/L), INR (1.35; 0.9–1.25), low fibrinogen (0.7 g/L; 1.7–4.2 g/L)) and a pancytopenia (platelets 103/nl, leukocytes 3.75/nl, hemoglobin 10.4 g/dL; full laboratory workup in Additional file 1: Table S1). SARS-CoV-2 PCR test, as well as blood and urine cultures, were negative. Abdominal ultrasound and echocardiography showed significant hepatosplenomegaly and a small pericardial effusion. The calculated HScore was 272 points indicating a very high probability (> 99%) for MAS [10].

Treatment with methylprednisolone (250 mg/d intravenously) and intravenous immunoglobulins was started, and anakinra was increased to 3 × 100 mg/d subcutaneously. Initial treatment response was moderate with reduced but remaining fever, decreasing ferritin levels, and normalizing cell counts after four days. She eventually received additional ciclosporin to achieve stable remission (2 × 100 mg/d). After 2 weeks, she could be discharged with her previous anakinra dose, ciclosporin and a steroid tapering regimen.

Despite her immunosuppressive treatment, the first vaccination had shown positive antibody development against SARS-CoV-2. Her planned second vaccination with BNT162b2 was interdisciplinary discussed, but eventually we recommended to wait for synthetic protein-based vaccine (e.g. NVX-CoV2373 (protein-based vaccine, Novavax)) to be approved by regulators, in the hope for a less inflammatory response.

Follow-up over a period of 4 month showed disease control and no clinical signs of relapse. Maintenance therapy during this period consisted of prednisolon (5 mg/d), anakinra (2 × 100 mg/d) and ciclosporin (2 × 100 mg/d). In a further step we plan to taper the ciclosporin to a reduced dose (2 × 50 mg/d).

## Discussion and conclusions

The new mRNA vaccines have generally shown a very reassuring safety profile. Nevertheless, it has been shown that nucleic acids in general, including mRNA, may become a target of pattern recognition receptors (PRR) [11]. Also, mRNA vaccines have been shown to act as pathogen-associated molecular patterns that activate toll-like receptors (TLR)-3–7 and -8 [12]. Stimulation of subsequent RNA sensors, such as RIG-I, MDA5 and OAS result in type I interferon (IFN) answer, which leads to maturation of dendritic cells, upregulation of MHC (major histocompatibility complex)-I and MHC-II as well as cytokine and chemokine expression [12–14]. Importantly, under type I IFNs, NK cells enhance IFN-γ production, which among many effects further stimulates macrophage activity [14]. In our patient a systemic type I IFN activation could be verified with significantly elevated CD169/SIGLEC1, a surrogate biomarker for type I IFN activity established in systemic lupus erythematosus, other rheumatic diseases and viral infections [15] 16. Overall, the new mRNA have been designed to provoke a broad immunological reaction including the innate and adaptive immune system [12]. One assumption might be that while protein-based vaccines tend to stimulate more B cellular pathways, those requiring a mRNA passage before the expression of the protein may stimulate more significantly also other cellular and inflammatory pathways involved in MAS. However, the exact mechanism by which type I IFN triggers MAS in our patient cannot be answered in detail and remains a hypothesis. In a majority of people vaccination leads to the anticipated effective production of antibodies and controlled T cell response without development of disease flares or induction of other autoimmune responses [17]. In few exceptions, such as in immune systems prone to highly inflammatory responses, also mRNA vaccines might act as trigger in MAS development.

To the best of our knowledge, this is the first case report of development of MAS in a patient with preexisting AOSD after vaccination in general, and SARS-CoV-2 vaccination in particular. Rare vaccine complications, especially in patients with autoimmune or autoinflammatory conditions are important to monitor and register. However, they do not diminish the overall very good safety profile and overwhelming positive benefit-risk ratio of licensed COVID-19 vaccines. Patients with rheumatic and autoimmune diseases are strictly advised to receive a SARS-CoV-2 vaccination, but should be carefully followed-up for signs of autoimmune complications. It remains difficult to establish causality in the case of rare adverse events, especially with preexisting conditions, and more studies are needed to address the incidence of specific autoimmune events following COVID-19 mRNA vaccination.

In a final statement we would like to underline the high safety and efficacy of SARS-CoV-2 vaccines in general to avoid any misinterpretations of this case report. It is crucially important for societies to attain a high vaccination rate for further control of the worldwide COVID-19 pandemic.

## Supplementary Information

**Additional file 1**. Full laboratory workup.

## Data Availability

The datasets used and/or analysed during the current study are available from the corresponding author on reasonable request.

## Abbreviations

AOSD: Adult-onset Still’s disease
MAS: Macrophage activation syndrome
PCR: Polymerase chain reaction
sIL-2-R: Soluble interleukine 2 receptor
LDH: Lactate dehydrogenase
IFN: Interferone
CRP: C reactive protein
TLR: Toll-like receptors
RIG-I: Retinoic acid-inducible gene I)
MDA5: Melanoma differentiation-associated protein 5
OAS: Oligoadenylate synthetases
MHC: Major histocompatibility complex
