# Supplementary material for: Macrophage activation syndrome in a patient with adult-onset Still’s disease following first COVID-19 vaccination with BNT162b2
Source: BMC Rheumatol. 2021 Dec 28;5:60. doi: 10.1186/s41927-021-00237-9 (PMC8712099; doi:10.1186/s41927-021-00237-9)
Supplement: Supplementary file 1 — Additional file 1. Full laboratory workup. [file 41927_2021_237_MOESM1_ESM.docx]

**Supplementary table 1:** Full laboratory workup

| **Parameter** | **Dimension** | **Reference** | **Admission** |
| --- | --- | --- | --- |
|  |  |  |  |
| Blood status |  |  |  |
| Hemoglobin | g/dl | 12.0-15.6 | **10.4** |
| Hematokrit (l/l) | l/l | 0.355-0.455 | **0.294** |
| Erythrocytes | /pl | 3.9-5.2 | **3.4** |
| Leukocytes | /nl | 3.90-10.50 | **3.75** |
| Thrombocytes | /nl | 150-370 | **103** |
| MCV | fl | 80.0-99.0 | 87.2 |
| MCH | pg | 27.0-33.5 | 30.9 |
| MCHC | g/dl | 31.5-36.0 | 35.4 |
| MPV | fl | 7.0-12.0 | 13.4 |
| RDW-CV | % | 11.5-15.0 | **15.7** |
| Rod-like neutrophiles | % | 0.5-10.0 | 4.2 |
| Segmented neutrophiles | % | 40.0-70.0 | **27.1** |
| Lymphocytes | % | 20.0-44.0 | 40.6 |
| Reactive lymphatic cells | % |  | 21.9 |
| Plasmacells | % | 0.0-1.0 | 1.0 |
| Monocytes | % | 2.0-9.5 | 5.2 |
| Eosinophiles | % | 0.5-5.5 | **0.0** |
| Basophiles | % | 0.0-1.8 | 0.0 |
| Immature thrombocytes | % | 1.1-6.1 | **11.6** |
| Erythroblasts | /nl | < 0.01 | 0.01 |
|  |  |  |  |
| Sodium | mmol/l | 136-145 | **130** |
| Potassium | mmol/l | 3.4-4.5 | 3.7 |
| Calcium | mmol/l | 2.15-2.50 | 2.17 |
| Calcium (corrected) | mmol/l |  | 2.37 |
| Phosphate | mmol/l | 0.87-1.45 | 1.15 |
| Osmolality (calculated) | mosmo/kg |  | 270 |
| Glucose | mg/dl | 74-106 | 104 |
| Creatinin | mg/dl | 0.50-0.90 | 0.88 |
| eGFR |  |  | >90 |
| Urea | mg/dl | 17-48 | 34 |
| Bilirubin, total | mg/dl | < 1.20 | **3.84** |
| Bilirubin, direct | mg/dl | < 0.30 | **3.60** |
| Bilirubin indirect | mg/dl | 0.00-0.75 | 0.24 |
| ALAT | U/l | < 31 | **207** |
| ASAT | U/l | < 35 | **659** |
| ALP | U/l | 35-105 | **1145** |
| gamma-GT | U/l | 5-36 | **385** |
| GLDH | U/l | < 4.8 | **54.4** |
| Lipase | U/l | 13-60 | 32 |
| Creatinkinase (CK) | U/l | < 167 | 73 |
| CK-MB | U/l | < 24.0 | **28.0** |
| CK-MB% | % |  | 38 |
| Troponin T hs | ng/l | < 14 | < 3 |
| Albumin | g/l | 35.0-52.0 | **32.1** |
| Protein | g/l | 64-83 | 79 |
| CRP | mg/l | < 5.0 | **46.4** |
| PCT (procalcitonin) | µg/l | < 0.50 | 0.31 |
| C3-Complement | mg/l | 900-1800 | 970 |
| C4-Complement | mg/l | 100-400 | 280 |
| Myoglobin | µg/l | 25-58 | **24** |
| Ferritin | µg/l | 13.0-150.0 | **136680** |
| HDL-Cholesterin | mg/dl | > 45 | **19** |
| LDL-Cholesterin | mg/dl | < 130 | 35 |
| Triglycerides | mg/dl | < 200 | **352** |
| LDH | U/l | 135-250 | **3190** |
| Free hemoglobin | mg/dl | < 2.00 | 26.00 |
| Haptoglobin | g/l | 0.30-2.00 | **<0.10** |
| Soluble transferrin receptor | mg/l | 1.9-4.4 | 2.7 |
| Soluble IL-2 receptor | IU/ml | < 710.0 | **14068** |
| Serum calprotectin (S100A8/9) | µg/ml | <2.94 | **>24** |
| Amyloid A Protein | mg/l | <6.4 | **16.9** |
| TSH | mU/l | 0.50-4.30 | 1.63 |
|  |  |  |  |
| Quick (TPZ) | % | 78-123 | **53** |
| INR |  | 0.90-1.25 | **1.35** |
| aPTT | sek. | 25.0-38.0 | 35.4 |
| D-Dimers | mg/l | < 0.50 | **>35** |
| Fibrinogen | g/l | 1.70-4.20 | **0.72** |
|  |  |  |  |
| Granulocytes absolute | /nl | 3.00-6.50 | **1.17** |
| Granulocytes relative | % | 50-80 | 31 |
| Lymphocytes absolute | /nl | 1.50-3.00 | 2.25 |
| Lymphocytes relative | % | 20-40 | **60** |
| Monocytes absolute | /nl | < 0.80 | 0.27 |
| Monocytes relative | % | 2-10 | 7 |
| NK cells absolute | /nl | 0.10-0.40 | **0.05** |
| NK cells, % of lymphocytes | % | 5-25 | **2** |
| CD19+ B-cells, % of lymphocytes | % | 5-25 | **3** |
| CD4/CD8 ratio |  | 1.1-3.0 | **0.3** |
| CD8-CD4-T-cells, % of T-cells | % | < 15.00 | 5.67 |
| CD8+CD4+-T-cells, % of T-cells | % | < 10.00 | 0.18 |
| CD3+ T-cells absolute | /nl | 0.90-2.20 | 2.12 |
| CD3+ T-cells, % of lymphocytes | % | 60-85 | **94** |
| CD4+ T-cells absolute | /nl | 0.50-1.20 | **0.43** |
| CD4+ T-cells, % of lymphocytes | % | 30-60 | **19** |
| CD4+ % T-cells | % |  | 20.34 |
| CD8+ T-cells absolute | /nl | 0.30-0.80 | **1.57** |
| CD8+ T-cells, % of lymphocytes | % | 20-40 | 70 |
| CD8+ % T-cells | % |  | 73.80 |
| Monocytic HLA-DR expression | AG/cell | > 15000 | 66054 |
| CD19+ B-cells absolute | /nl | 0.10-0.40 | **0.07** |
| CD169/SIGLEC1 expression | AG/cell | < 2400 | **5753** |
|  |  |  |  |
| Nasal swab: |  |  |  |
| SARS-CoV-2 (PCR) |  |  | **negative** |
|  |  |  |  |
| Antibodies (after 1 vaccination): |  |  |  |
| SARS-CoV-2 (S-Ag) IgG (Elisa: Elecsys ^®^ Anti-SARS-COV-2) |  |  | **positive** |
| SARS-CoV-2 (S-Ag) IgA (Elisa: Elecsys ^®^ Anti-SARS-COV-2) |  |  | **positive** |
